# Probing the affinity of noble metal nanoparticles to the segments of the SARS-CoV-2 spike protein

**DOI:** 10.1016/j.isci.2023.106110

**Published:** 2023-02-04

**Authors:** Qiuyang Lu, Baiyang Zhang, Mingzi Sun, Lu Lu, Baian Chen, Hon Ho Wong, Cheuk Hei Chan, Tong Wu, Bolong Huang

**Affiliations:** 1 Department of Applied Biology and Chemical Technology, The Hong Kong Polytechnic University, Hung Hom, Kowloon, Hong Kong, China; 2 Chinese International School, Hong Kong, Hong Kong, China; 3 Research Centre for Carbon-Strategic Catalysis, The Hong Kong Polytechnic University, Hung Hom, Kowloon, Hong Kong SAR, China

**Keywords:** chemical property, simulation in materials science, analytical method in materials science

## Abstract

Currently, scientists have devoted great efforts to finding effective treatments to combat COVID-19 infections. Although noble metal nanoparticles are able to realize protein modifications, their interactions with the protein are still unclear from the atomic perspective. To supply a general understanding, in this work, we have carried out theoretical calculations to investigate the interaction between protein segments (RBD1, RBD2, RBD3) of SARS-Cov-2 spike protein and a series of noble metal (Au, Ag, Cu, Pd, Pt) surfaces regarding the binding strength, protein orientations, and electronic modulations. In particular, the Au surface has shown the strongest binding preferences for the protein segments, which induces electron transfer between the Au and receptor-binding domain (RBD) segments. This further leads to the polarization of segments for virus denaturation. This work has offered a direct visualization of protein interactions with noble metal surfaces from the atomic level, which will benefit anti-virus material developments in the future.

## Highlights

• Probing of interactions between noble metals and spike protein segments
• Demonstrate the electron transfer from proteins to the Au surface after binding
• Visualize the polarization effects of protein segments on noble metal surfaces

## Graphical abstract

**Figure:**
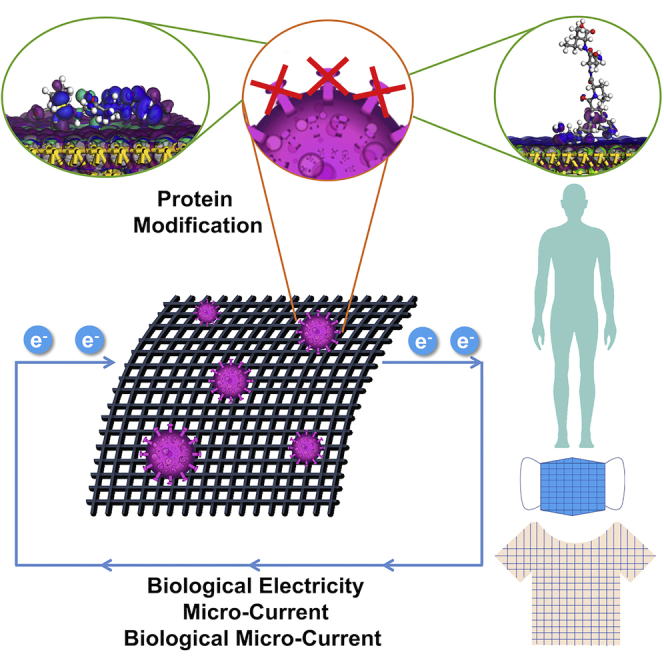

## Introduction

It has been about three years since the initial outbreak of coronavirus disease 2019 (COVID-19) in December of 2019 inducing more than 6 million deaths. A large amount of affordable and effective prevention methods and vaccines have emerged to control the COVID-19 pandemic however the recently reported data suggest more than one wave of outbreak sweeping across the world due to the Omicron variants, and the clinic system of each country faces great challenges and experiences difficulties. Although contemporary methods for combating COVID-19, such as vaccinations and social distancing have been demonstrated to sufficiently restrain the pandemic but confirmed cases intensively increase because the quest for effective treatment is still open and the population with immunodeficiency or chronic diseases is extremely dangerous when exposed to the virus. It inspires more studies for sufficient and feasible methods to provide substantive protection to the catastrophic pandemic. COVID-19 is caused by the virus identified as SARS-CoV-2 and its peripherally composed proteins exhibit a solar corona-like shape (Figure S1A). The single-stranded RNA virus is comprised of approximately 30,000 nucleotides with nucleotide lengths ranging from 27 to 32 Kilobytes (KB).^1^ Among them, spike protein (S protein) mediates viral entry by binding to the cell receptor angiotensin-converting enzyme 2 (ACE2) (Figure S1B). The receptor-binding domain (RBD) localizing on S protein can specifically recognizes ACE2 and enable viral entry. Based on the pathogenesis and clinical features of patients with COVID-19, researchers have demonstrated the efficiently increased immune evasion and infectivity of SARS-CoV-2 compared to that of SARS-CoV due to the mutation of S protein. Since the beginning of 2022, the comparatively milder SARS-CoV-2 variant Omicron has started to rise as the dominant strain at the time, featuring lower viral load and slighter symptoms.^2^ However, it also triggers a long discussion about if the continuous antigenic evolution of the virus can lead to a divergent variant characterizing both higher reinfection rate and disease severity. RBD is located on the S1 subunit (Figure S1C) of the S protein and binds with the host cellular receptor ACE2 for viral entry.

Meanwhile, noble metals have shown impressive performances in catalysis due to their strong capability in adsorption and electron transfer with molecules. Such features lead to significant advances in previously inaccessible organs and tissues, as well as further applications in the delivery of drugs, antigens, and other chemical compounds.^2^^,^^3^ Moreover, noble metals have also been widely applied in biological detection and characterization.^4^^,^^5^^,^^6^^,^^7^ Especially for detecting the virus protein including H1N1, and SARS-CoV-2 spike protein, noble metal nanoparticles have shown high detecting sensitivity by combining with other nanostructures.^8^^,^^9^^,^^10^^,^^11^ Owing to the frequent utilization of noble metals for industrial and medical purposes, their versatile and complex characteristics have also stimulated numerous studies and particular experimental methods. In previous studies, it has been shown that Au, Ag, Cu, Pd, and Pt nanoparticles have a strong binding affinity for cellular receptors.^12^^,^^13^^,^^14^ Because of their strong ability in binding proteins and the consequent structural reorganization, the Au nanoparticles also have been proven to efficiently inhibit not only the folding of Aβ and Tau protein of Alzheimer’s disease but also herpes simplex viruses, C virus, and HIV infection.^15^ The Au surface has been sufficiently investigated and widely utilized in self-assembled nanosystems.^16^^,^^17^^,^^18^ More importantly, Au has also been a mainstay of biology and medical science for a long time featuring high stability and low toxicity. In addition, the Au particles also can be easily surface-modified with a variety of molecules, such as proteins, antibodies, and peptides to improve their efficiency and stability.^19^ Although the great potential of noble metals has been widely proven in biomedical fields, their applications in COVID-19-related clinic treatments are still very limited.

**Figure 1:**
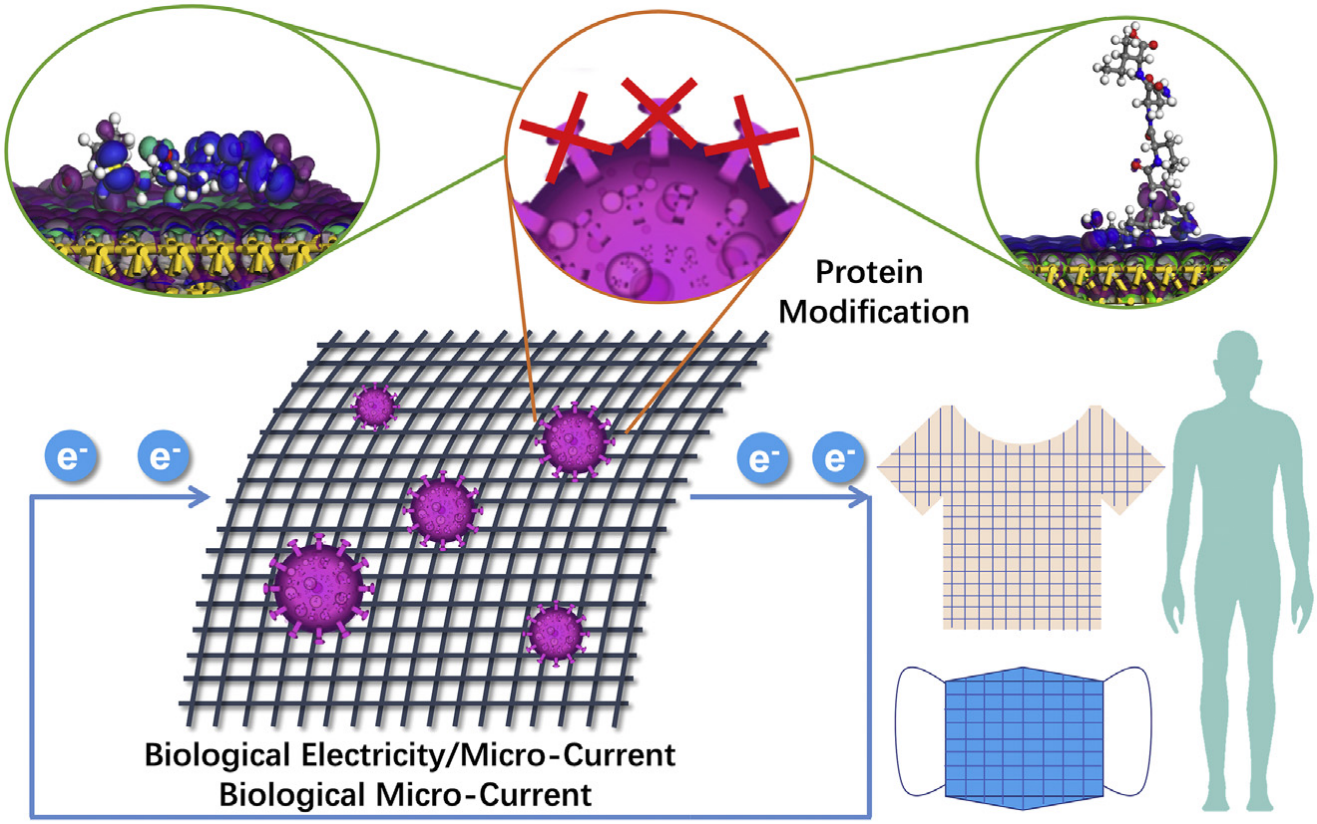
Schematic diagram of the proposed noble metal designed for anti-virus applications such as masks and filters

Therefore, in this work, we present a theoretical strategy to explore the interaction behaviors between noble metals and key RBD segments of the S protein in the SARS-CoV-2 virus. We have evaluated the binding preferences between the RBD segments and diverse noble metal surfaces. By utilizing the density functional theory (DFT) calculations, we have established discrete models featuring the amino acid sequences on the RBD site. The influences of the binding interactions on the electronic configurations of the RBD segments are examined to determine the possibility of protein denaturation. Our results indicate that the interactions between noble metals and proteins are guided by the potential difference. The Au surface has been demonstrated to be the most proactive affinity for binding selected RBD segments, and the horizontal structural configurations expose more active sites to promote strong interactions. Our theoretical calculations have offered significant insights into the specific interaction mechanism between protein segments and noble metal surfaces. This work supplies preliminary guidance on the design and synthesis of specific noble metals as a broad-range antiviral material for disinfectants including masks and filters, which opens the possibility of implementing noble metals as practical tools for mitigating SARS-CoV-2 and future pandemics (Figure 1).

## Results

RBD is located on the S1 subunit of the S protein and binds with the host cellular receptor ACE2 for viral entry (Figure S2). For this preliminary exploration, we considered that the interaction between the surface and the protein is affected by mainly two factors: the noble metal selections and the contacting site on the protein segment. Thus, we choose the short protein segments as the modeling target instead of a single amino acid as reported by previous research works.^20^^,^^21^ The RBD-ACE2 interaction also relies on specific residues and the bonding between the residues or molecules is capable of changing the virus property.^22^^,^^23^ We have selected amino acid segments from the RBD segments as the theoretical models in our work, where similar methods have been reported in previous studies.^12^^,^^24^^,^^25^ To observe the interactions from the atomic level, we specifically picked out 3 segments with 5 amino acids, which are located on the front side of the RBD (Figure S3). Here, considering the calculation loading challenge, we excluded the consideration of the secondary and tertiary structure and only select a short length of target segments. Due to the complexity of the protein adsorption progress, we have selected five noble metal (Pt, Pd, Cu, Ag, Au) surfaces to discuss the possibility of binding protein segments, which can further guide the exploration of the design and modification of the noble metal particles as antiviral equipment.

**Figure 2:**
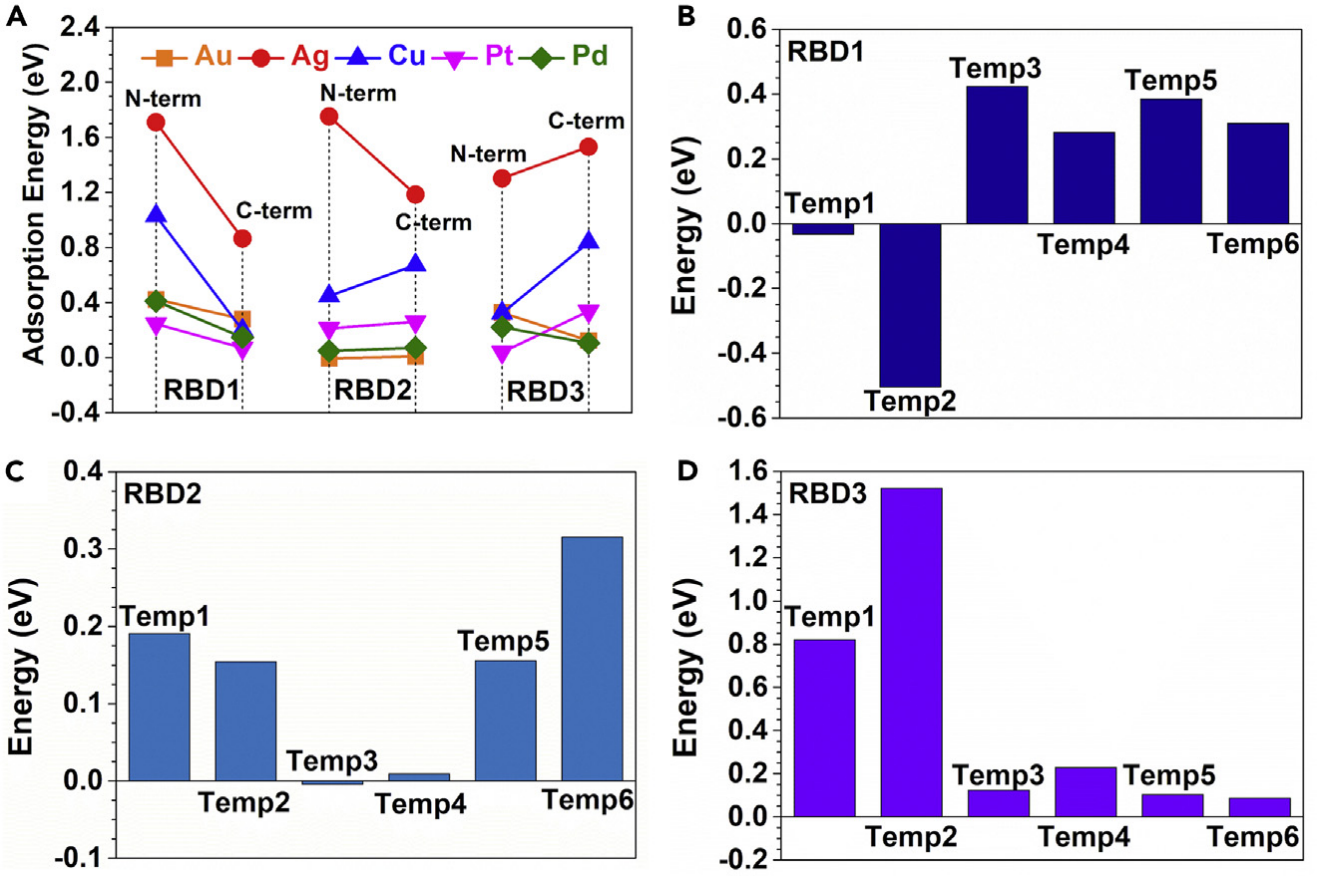
Adsorption behaviors of RBD segments (A–D) Adsorption energy comparison of different RBD segments on different metal surfaces. N-term and C-term represent the binding configurations. Adsorption energy comparison of (B) RBD1, (C) RBD2, and (D) RBD3 segments on Au (111) surfaces with different binding configurations.

To better analyze the difference between metal species when contacting with protein segments, we have applied two different binding configurations for each segment on each metal surface. The two configurations are the N- and C-terminal of each protein segment. It can be observed that different RBD segments exhibit different levels of adsorption energy (Figure 2A). For RBD segments in general, being adsorbed onto the Ag and Cu surfaces display the highest adsorption energies, which indicate an unstable state. In comparison, Pt, Pd, and Au show relatively low energy costs for RBD segment adsorption, supporting a more stable adsorption state. For RBD1 segments, we noticed that Pt surfaces show the strongest binding with the lowest adsorption energy levels of approximately 0.1 eV in the C-terminal. Moreover, contrary to the previous hypothesis, all metals adsorbed with the RBD1 configuration display greater binding affinity and adsorption stability with the C-terminal than with the N-terminal. However, in the RBD2 segments, some metals such as Cu, Pt, Pd, and Au had a slightly more stable configuration with the N-terminal rather than the C-terminal. Notably, the configuration where the RBD2 N-terminal is adsorbed with the Au surface presents the lowest adsorption energy of approximately 0 eV, which indicates Au as a particularly stable adsorption surface relative to the other elements. For the RBD3 segments, the adsorption energy configurations are similar to that of RBD2 where certain elements, notably Ag, Cu, and Pt have more stable binding configurations with the N-terminal due to lower adsorption energies as compared to the C-terminal adsorption energies of the respective elements. However, Au and Pd in RBD3 show more stable configurations with the C-terminal, which further reveal that noble metal surfaces do not universally have more stable adsorption bonds with the N-terminal binding configurations. Previous experiments have demonstrated strong Au-S bonds between Au substance and cysteine by scanning probe microscopy,^18^ which reached up to 2.0 eV and led to the conformational change of both surface and protein. The adsorption energy of Au-S varies in the range from 0.13 eV to 2.0 eV when the binding environment changes.^26^^,^^27^^,^^28^^,^^29^ Compared with experiments, the strongest binding energy obtained from the DFT method is around 1.5 eV, which is fully sufficient to support the potential structural denaturation of the RBD segments. Although our calculation results only consider the binding sites and surface environments, the overall adsorption energies are in a similar range to the experiments. In addition, the concave on the RBD segment sites can also demonstrate the strong ability of Au surface denaturing protein segments.

In addition, we further compared the binding energies of the S-terminal configurations for RBD2 on different noble metal surfaces (Figure S4). Notably, there are no evident advantages for the S-terminal binding configurations. For the Au surface, the binding energy of the S-terminal configuration is only 0.33 eV, which is still higher than both the C- and N- terminal configurations, leading to the weaker selectivity for S-terminal. Notably, only the Ag surface displays the strongest selectivity toward the S-terminal binding configurations. However, considering that all the binding energies are relatively high (> 0.9 eV), the Ag surface is not suitable for binding with RBD2 segments. For the Cu surface, the binding energy of the S-terminal configuration is much higher than that of the C- and N- terminal configurations, leading to very low preferences for such binding. For both Pt and Pd surfaces, we notice that different terminal binding configurations exhibit highly similar energy costs. In addition, the energy costs of these surfaces are small (< 0.5 eV), which suggests the possible binding of RBD2 on these surfaces. However, the selectivity of specific terminals is difficult to distinguish.

In the further assessment of the Au surface, we varied the contact configurations of each segment. Except for the vertical alignments of the N- and the C-terminal, we added 4 different models with horizontal binding configurations of the protein segments for providing more contact sites. The Temp2 binding configuration of RBD1 (Figures 2B and S5A) has shown a highly preferred binding trend with negative binding energies. This indicates that the binding and adsorption of RBD1 will occur spontaneously and form a stable bond with the Au surface. Moreover, the RBD1-Temp2 is a horizontal configuration, which does not exclusively bind the N- or C-terminal to the Au surface. Therefore, this result demonstrates that specific binding configurations such as the N- or C-terminal are not required to guarantee efficient binding between RBD segments and noble metal nanoparticle surfaces. We also noticed that even the highest binding energies of RBD1 are smaller than 0.5 eV, which indicates that the Au surfaces are still capable of adsorbing the RBD1 segments on all surfaces. However, the physiological environment contains several different proteins, and whether the binding affinity can be identified as the specific target of Au needs further assessment.

The binding of RBD2 in Figures 2C and S5B display overall lower adsorption energy as compared to RBD1, where the largest adsorption energy is 0.31 eV for RBD2-Temp6. This indicates that the RBD2 segment also has a high potential for successful and stable binding with the Au surface. The lowest adsorption energy for RBD2 is Temp3, where the RBD2 segment is vertically placed onto the surface with the N-terminal in contact with the Au surface, which leads to spontaneous adsorption with negative energy costs. This is different from RBD1, where RBD1-Temp3 with the highest adsorption energy also shows the N-terminal, indicating that the N-terminal preference is not universal. In contrast, the adsorption energy results for RBD3 (Figures 2D and S5C) are the highest since no negative adsorption energy can be achieved by contacting the surface with six different configurations. In particular, the most unfavored surface binding of RBD3-Temp2 reaches high adsorption energy of 1.55 eV. This shows a strong difference with the smallest adsorption energy of 0.08 eV for RBD3. Due to large variations of the adsorption energies, the binding of RBD3 with the Au surface mostly depends on the binding sites in specific binding configurations. These adsorption energy comparisons have revealed that the modification of the protein is highly related to the RBD segments, where different binding preferences will lead to the varied possibility of modulations.

Based on the initial binding energies of RBD segments, Pd and Pt surfaces have also exhibited low energy costs, which potentially show high affinity with the protein. For further understanding of the binding behavior, we have demonstrated the binding energies of RBD1-RBD3 segments on the Pd surface (Figures S6A–S6C). It is noted that all the binding energies are overall much higher than that on the Au surface. For RBD1, only limited binding configurations show small energy costs. Although certain binding configurations of RBD2 exhibit very high energy costs, most of the binding energies are smaller than 0.5 eV, leading to possible binding on the Pd surface. Among the three segments, RBD3 is the most energetically unfavored for binding due to the largest average energy costs. Meanwhile, we notice similar binding trends of RBD segments on the Pt surface (Figures S6D–S6F). RBD1 segments display the weakest sensitivity to the binding configurations, where the binding energy changes are mildest. However, for the RBD2 segment, it is noted the evidently stronger selectivity to the Temp1 and Temp4-6 binding configurations due to the much lower energy costs (< 0.5 eV) than other binding configurations. Similar to the Pd surface, the RBD3 shows overall higher energy costs than RBD1 and RBD2 on the Pt surface, supporting the weakest binding preference on noble metal surfaces.

**Figure 3:**
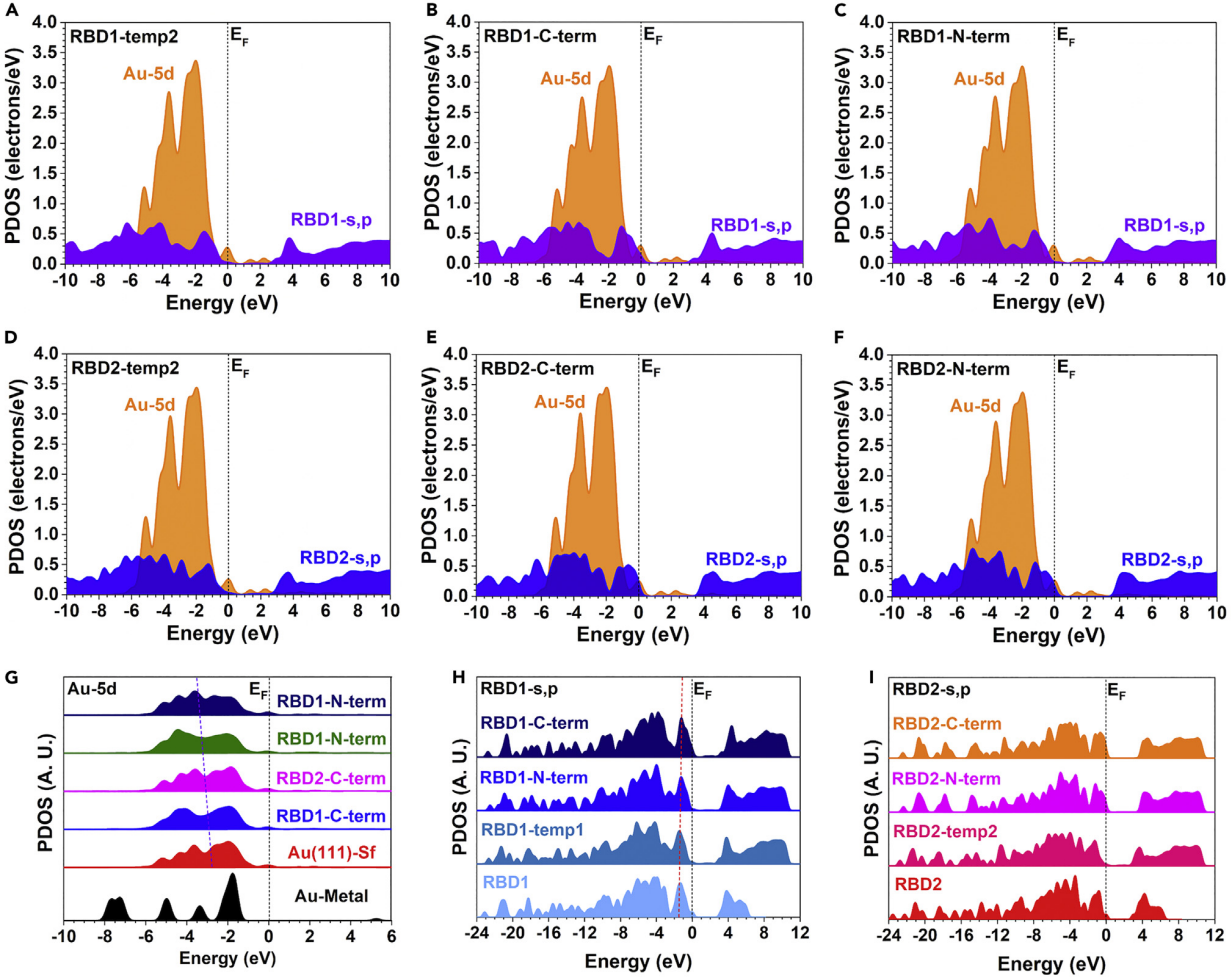
Electronic modulations of interactions between Au surface and RBD segments (A–C) PDOS of Temp2 binding configuration for RBD1 on Au surface. PDOS of (B) C-terminated and (C) N-terminated binding configurations for RBD1 on Au surface. (D–F) PDOS of Temp2 binding configuration for RBD2 on Au surface. PDOS of (E) C-terminated and (F) N-terminated binding configurations for RBD2 on Au surface. (G) Site-dependent PDOS of Au-5*d* in different binding configurations. (H) Site-dependent PDOS of RBD1-*s*,*p* orbitals in different binding configurations. (I) Site-dependent PDOS of RBD2-*s*,*p* orbitals in different binding configurations.

Then, we further investigated the interactions between the RBD segment and noble metals from the perspectives of electronic structure regarding different binding configurations, which are demonstrated by the projected density of states (PDOS). As a representative, we have selected the Au surface to reveal the electronic modulations induced by the interactions with protein segments. For RBD1, we have compared three different binding configurations as shown in Figures 3A–3C. For the most stable binding configuration (Temp2), we noticed that the *s*,*p* orbitals of RBD1 have a broad distribution crossing the Fermi level (E_F_). Meanwhile, Au-*5d* orbitals show a strong peak at E_V_-2.0 eV (E_V_ = 0 eV), which represents the most electroactive orbitals to interact with the surface-adsorbed RBD segments. We have noticed the evident overlapping between the Au and RBD orbitals, which indicates empirical evidence to support the efficient *s-p-d* couplings, and the tight adsorption. For both N-terminated and C-terminated binding configurations, we did not find the evident change of both Au and the RBD segments, which represents that the electron transfer between the Au and the RBD segments still exists. For RBD 2, shown in Figures 3D and 3E, no evident change has been induced when compared to RBD1. We only notice that the *s*,*p* orbitals of RBD2 in N-terminated and C-terminated binding configurations show slightly closer distances to the E_F_ than that in the Temp2 configurations. This indicates that the *s*,*p* orbitals may be modified after binding with the Au surface.

Figures 3F and 3G has shown the electronic structure of the RBD and different Au sites. After binding with RBD segments, we have noticed that the flat Au surfaces have become slightly concave at the binding sites, which indicates the strong binding phenomenon can induce configurational variation. Compared to the bulk Au metal and pristine surface, the adsorption of RBD segments has resulted in the downshifting of Au-5*d* orbitals toward lower positions, which indicates that the Au surface has become more electron-rich at the binding sites, supporting the electron transfer from the RBD. Moreover, the electron transfer trends of N-terminated binding configurations are stronger than the C-terminated binding configurations. These results prove the interactions between local Au surfaces and the RBD segments. In contrast, the *s*,*p* orbitals of RBD1 have shown a converse evolution change to the Au-5*d* orbitals (Figure 3G). This further confirms that RBD segments lose electrons toward the Au surface, especially for RBD1. Although the upshifting trend of RBD2 is not as evident as RBD1, we still find that the *s*,*p* orbitals are approaching the E_F_ in Figure 3H, which also supports the electron-losing trends in the segments. Therefore, through the opposite electronic structure evolution trends of Au and RBD segments, we have confirmed that the electron transfer induced by the orbital couplings between Au-5*d* and RBD-*s*,*p* orbitals.

**Figure 4:**
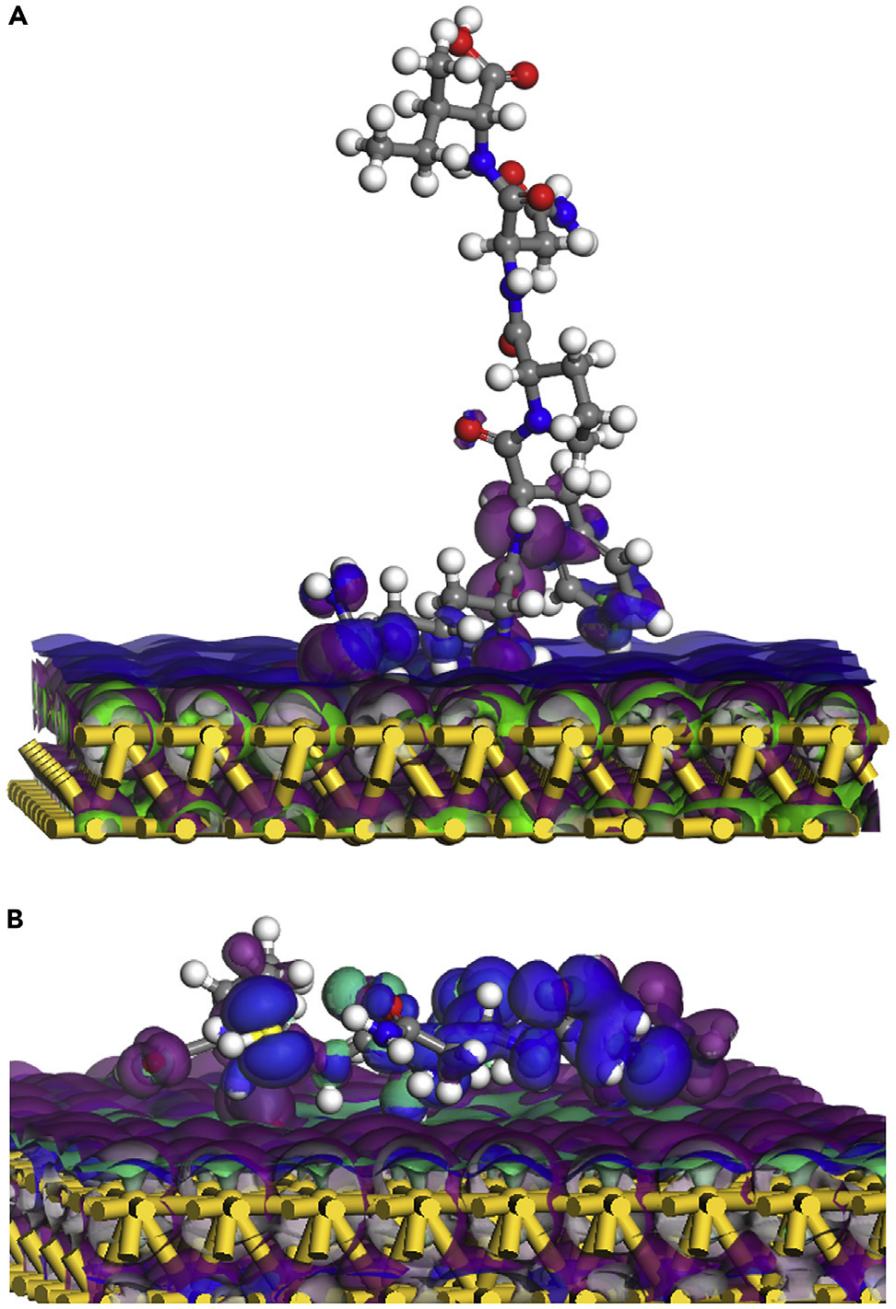
Visualizations of orbital coupling between Au surface and RBD segments (A and B) 3D contour plots of the electronic distributions in (A) RBD1-Temp3 and (B) RBD2-Temp1 on the Au surface. The green isosurface represents the anti-bonding orbitals and the blue isosurface represents the bonding orbitals. The purple isosurface represents the orbital couplings of Au and RBD segments. Gold balls = Au, Grey balls = C, Blue balls = N, Red balls = O, and White balls = H.

To illustrate the interactions between the Au surface and the RBD segments, we have visualized the electronic distributions of the adsorbed RBD segments with different binding configurations shown in Figure 4. We have selected three different models to demonstrate the modulations on the RBD segments due to the orbital coupling effect. The selected models are RBD1-Temp3 and RBD2-Temp1, which all display different binding configurations to reveal the electronic modulations. Notably, the bonding orbitals are noticed at the bonding regions between RBD1 and Au surface, revealing the strong bonding (Figures 4A and S5). The N-sites of RBD segments show strong bonding orbitals, which are induced by the *p*-*d* coupling with the Au surface. It is noticed that electronic modulations of the *p-d* couplings show a limited range since only the bottom of the RBD1 segment will be affected. In comparison, For RBD2-Temp1 binding, the RBD2 segments are horizontally placed on the Au surface, which shows multi-binding sites with the Au surface (Figure 4B). In contrast with the vertical binding, the parallel binding configurations show stronger modulations on the RBD segments. The bonding orbitals mostly concentrate on the RBD2 segments, which pull the bonding orbitals from the Au surface toward the RBD2. The anti-bonding orbitals are very weak, which unravels the existence of the *p*-*d* coupling effect. Moreover, we notice that the whole RBD2 segments have shown strong electronic distributions, indicating that the horizontal binding configurations potentially lead to stronger modifications on the protein. Most importantly, the *p*-*d* coupling effect has resulted in uneven electronic distributions, which represent the polarization in the RBD segments. With the strong polarization effect, the function of the RBD or even the S protein is highly possible to be modulated or even disabled.

We conclude that, during the process of surface binding between noble metal surfaces and the protein segments, the *d* shells of the noble metal surfaces would overlap with the *p* or *s-p* hybridized orbitals of the RBD segments to initiate electron transfer from the segments to the metal surfaces. This is because the metal surface contains a lower potential with empty *d* shells while the RBD segment contains more electrons and thus possesses a higher potential. Hence, we could predict that the electrons previously within the high potential *s-p* shells of the RBD segment will be transferred to the low potential orbital of the noble metal surface. Subsequently, the RBD segments lose electrons on the side bound to the metal surface, inducing a positively charged end. During the electron redistribution, the protein segments will be polarized hence the un-contacted side becomes a negatively charged end. We assume that the electrostatic attraction between the dipole and the surface will lead to configurational changes to the specific protein site, as the attraction will bend and reshape the bond structures of the RBD segment.

## Discussions

In this work, we have carried out theoretical explorations on the interactions between S protein segments and the noble metal surfaces including Au, Ag, Cu, Pt, and Pd, which have been applied in biomedical applications because of their low toxicity, stability, and electroactive nature. After comparisons, we have noticed that the binding preference of protein segments is strongly varied on different metal surfaces. In particular, Au, Pt, and Pd are the most viable candidates for stable adsorption with RBD segments. For different segments, RBD2 and RBD1 are the most stable binding segments of the S protein, which can be considered as the target segments in future experiments. Moreover, through the detailed investigations of the electronic structures in the different binding configurations, we have noticed the evident *s-p-d* coupling effect, where the electrons transfer from RBD toward the Au surface. Such an orbital coupling effect is the key to the strong interactions between Au and protein segments, which significantly promotes protein modifications. Moreover, from electronic distributions, we have clearly visualized the strong polarization of RBD segments induced by the binding with Au surfaces. The larger binding areas of the parallel bindings lead to a stronger polarization effect in RBD segments, which also potentially induces stronger protein modifications. Based on this work, we are able to obtain important information for understanding protein modifications and suppressions of the SARS-CoV-2 virus. Introducing noble metal nanoparticles as an effective means to disable the functionality of the S protein and hence prevent the entering of the virus into the human body. This may open a new route in disease treatments of the SARS-Cov-2 virus and benefit more research in this field.

### Limitations of the study

For the applications of noble metal nanoparticles in SARS-CoV-2 virus detection or treatment, the protein modifications happen in more complicated environments such as the human body. This usually involves many environmental factors including temperature, pressure, humidity, and pH value which will potentially change the properties of metal surfaces and proteins. However, for current DFT calculations, the consideration of these environmental factors is still a significant challenge, which also leads to massive requirements of computational loading. In the future, more efforts still need to consider these environmental factors in theoretical explorations. The molecular dynamic (MD) simulations can be considered to evaluate the protein binding at different temperatures and pressures. Meanwhile, introducing the water layers and other ions can also supply important references for understanding the practical applications of noble metal nanoparticles in the internal human body or solution environments.

## STAR★Methods

### Key resources table

**Table:** 

REAGENT or RESOURCE	SOURCE	IDENTIFIER
**Software and algorithms**		

Cambridge Serial Total Energy Package (CASTEP)	Clark et al.^30^	http://www.castep.org/

### Resource availability

#### Lead contact

Further information and requests for resources should be directed to and will be fulfilled by the lead contact, Bolong Huang (bhuang@polyu.edu.hk).

#### Materials availability

This study did not generate any new reagents.

### Methods details

#### Calculation method

To investigate the interactions between RBD segments and noble metal surfaces, we have applied the density functional theory (DFT) within the CASTEP codes to investigate the reaction treads and affecting factors.^30^ For all the calculations, we have selected the generalized gradient approximation (GGA) with the Perdew-Burke-Ernzerhof (PBE) to accurately reveal the electron interactions regarding the exchange-correlation energy.^31^^,^^32^^,^^33^ The 380 eV plane-wave cutoff energy with ultrafine quality has been used and the ultrasoft pseudopotential scheme has been applied for all the geometry optimizations.^34^ The convergence tests verify the cutoff energy selection and the Broyden-Fletcher-Goldfarb-Shannon (BFGS) algorithm is applied for the k-point mesh to achieve the energy minimization.^35^^,^^36^ All the noble metal surfaces have been cleaved from (111) surfaces with the three-atomic layer thickness in a 9 × 9 × 1 supercell. Meanwhile, we have also applied 30 Å vacuum space in the z axis to maintain adequate space for the relaxation of proteins. To guarantee the calculation accuracy, we have applied the following convergence criteria for the geometry optimizations: the Hellmann-Feynman forces should be less than 0.001 eV/Å; the total energy should not exceed 5 × 10^−5^ eV per atom.

## Data Availability

• All data reported in this paper will be shared by the lead contact upon request. • This paper does not report the original code. • Any additional information required to reanalyze the data reported in this paper is available from the lead contact upon request.
