# Supplementary material for: Probing the affinity of noble metal nanoparticles to the segments of the SARS-CoV-2 spike protein
Source: iScience. 2023 Feb 4;26(3):106110. doi: 10.1016/j.isci.2023.106110 (PMC9898944; doi:10.1016/j.isci.2023.106110)
Supplement: Document S1. Figures S1–S6 [file mmc1.pdf]

iScience, Volume *26*

Supplemental information

Probing the affinity of noble metal nanoparticles to the segments of the SARS-CoV-2 spike protein

Qiuyang Lu, Baiyang Zhang, Mingzi Sun, Lu Lu, Baian Chen, Hon Ho Wong, Cheuk Hei Chan, Tong Wu, and Bolong Huang

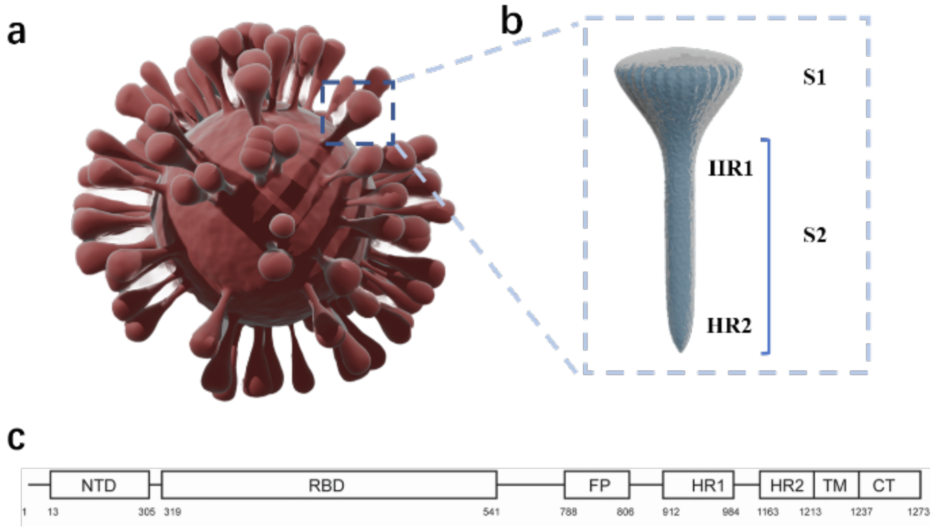

Figure S1. Demonstrations of the SARS-CoV-2 virus. Related to Figure 1.

(a) Schematic illustration of SARS-CoV-2 virus. (b) The S protein functions as the binding bridge to the receptor ACE2. (c) Schematic representation of the SARS-CoV-2 spike.[1]

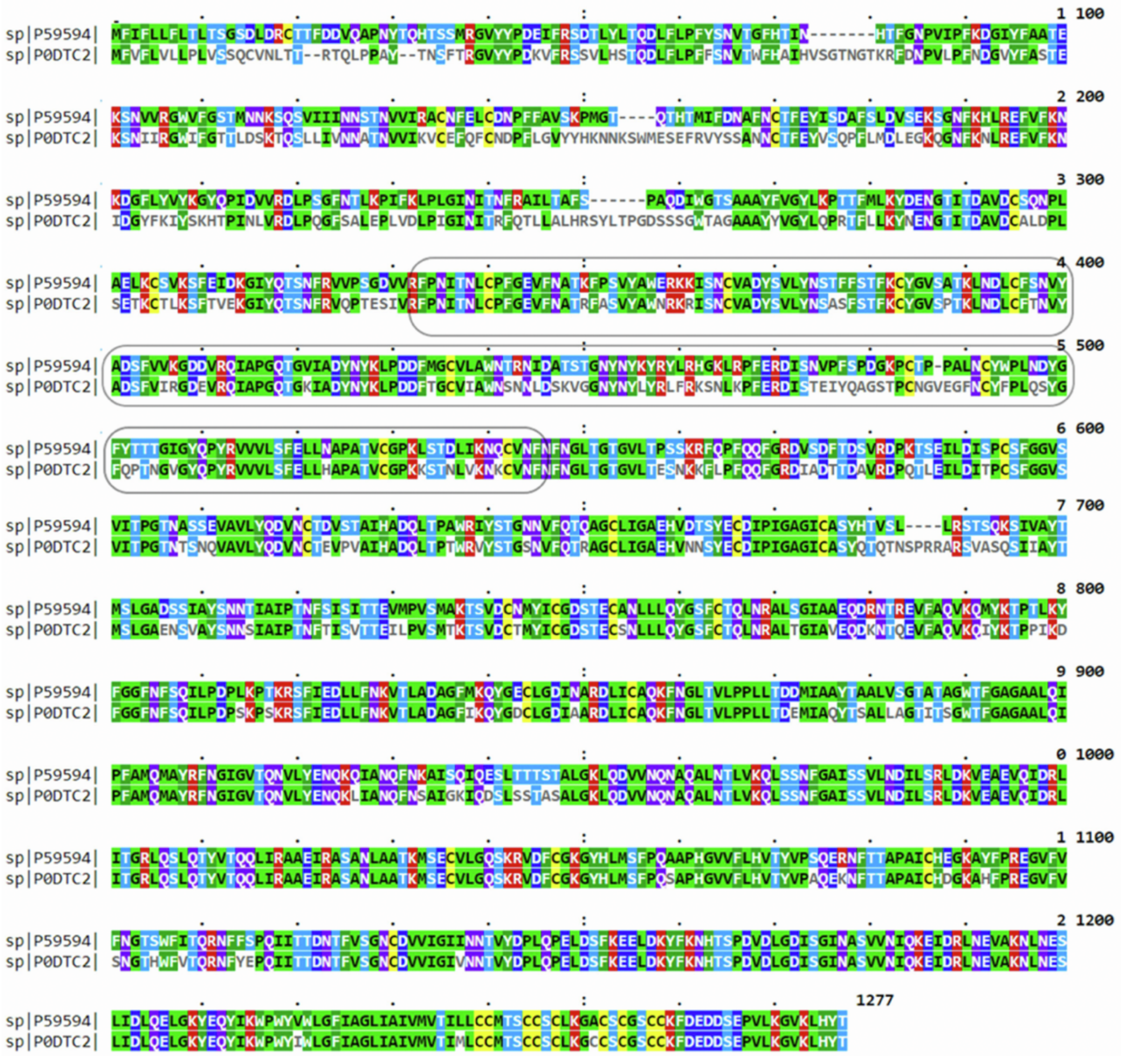

Figure S2. The complete multiple sequence alignments of the S protein of SARS-CoV-2.[2]
Related to Figure 1.

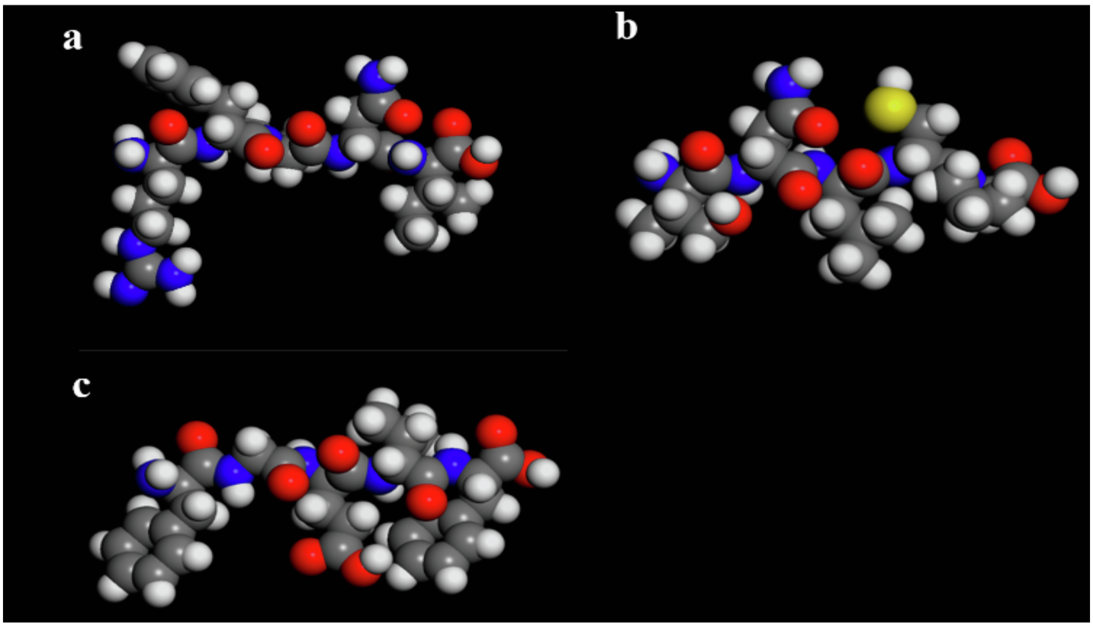

Figure S3. The simulated amino acid segments. Related to Figure 2.

(a) RBD fragment 1: RFPNI, contains amino acids Arginine, Phenylalanine, Proline, Asparagine, and isoleucine, the chemical formula is $C_{30}H_{47}N_9O_7$. (b) RBD fragment 2: TNLCP, contains amino acids Threonine, Asparagine, Leucine, Cysteine, and Proline, the chemical formula: $C_{23}H_{40}N_6O_8S$. (c) RBD fragment 3: FGEVF, contains amino acids Phenylalanine, Glycine, Glutamic Acid, Valine, and Phenylalanine, the chemical formula: $C_{30}H_{39}N_5O_8$. Grey balls = C, Blue balls = N, Red balls = O, Yellow balls = S, White balls = H.

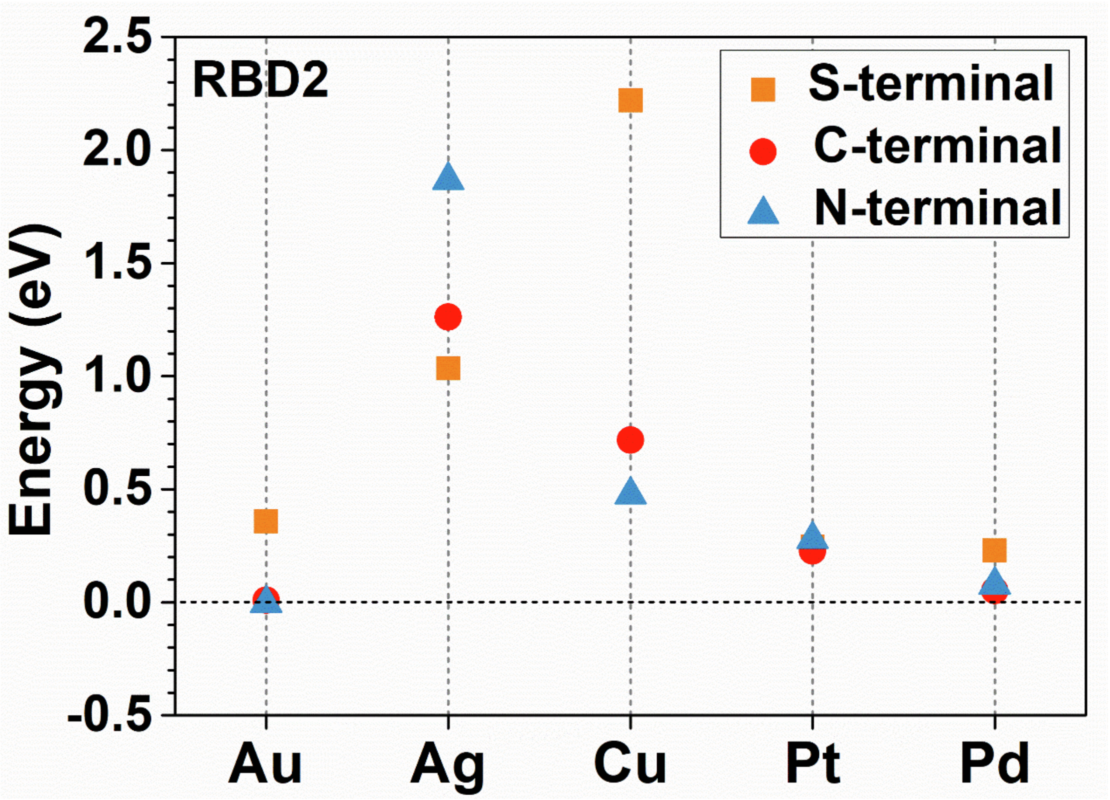

Figure S4. The binding energy comparisons of RBD2 in the S-terminal with other binding configurations. Related to Figure 2.

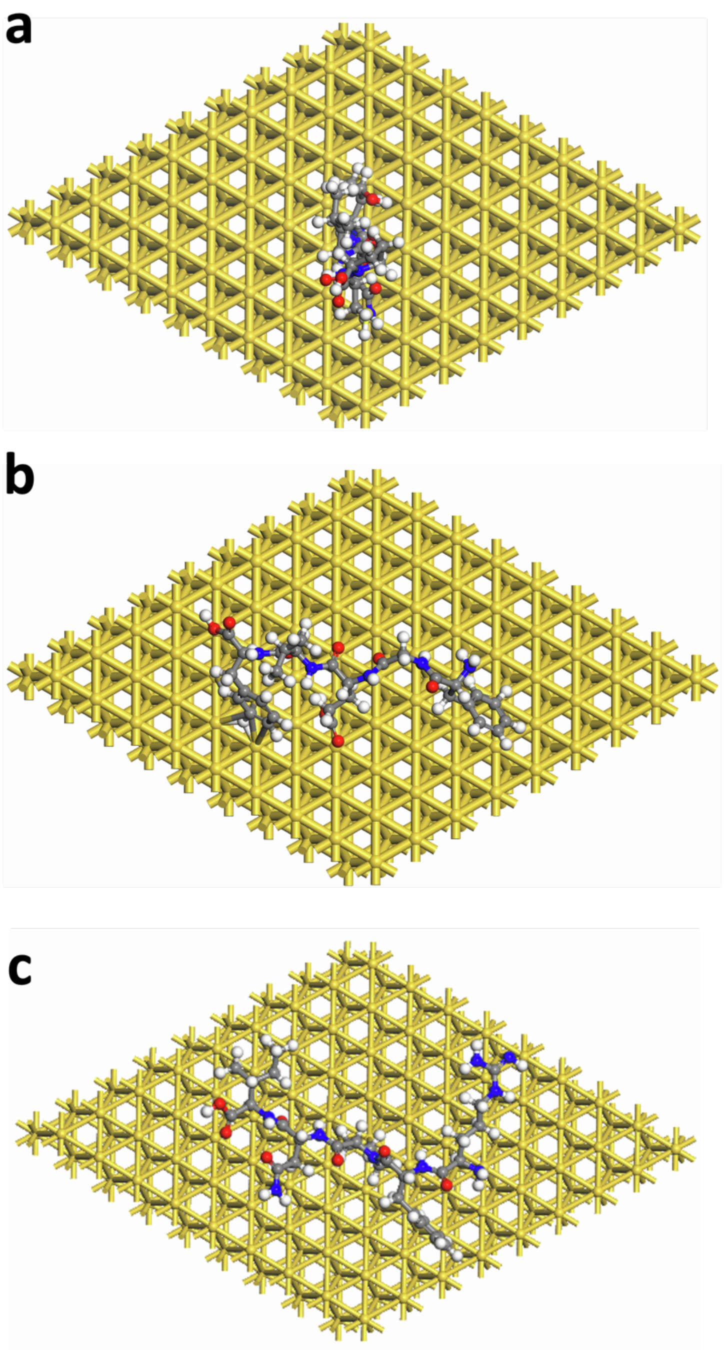

Figure S5. **Binding configurations of different RBD segments on the surface.** Related to Figure 2.

(a) of RBD1-Temp2, (b) RBD2-Temp3, and (c) RBD3-Temp6.

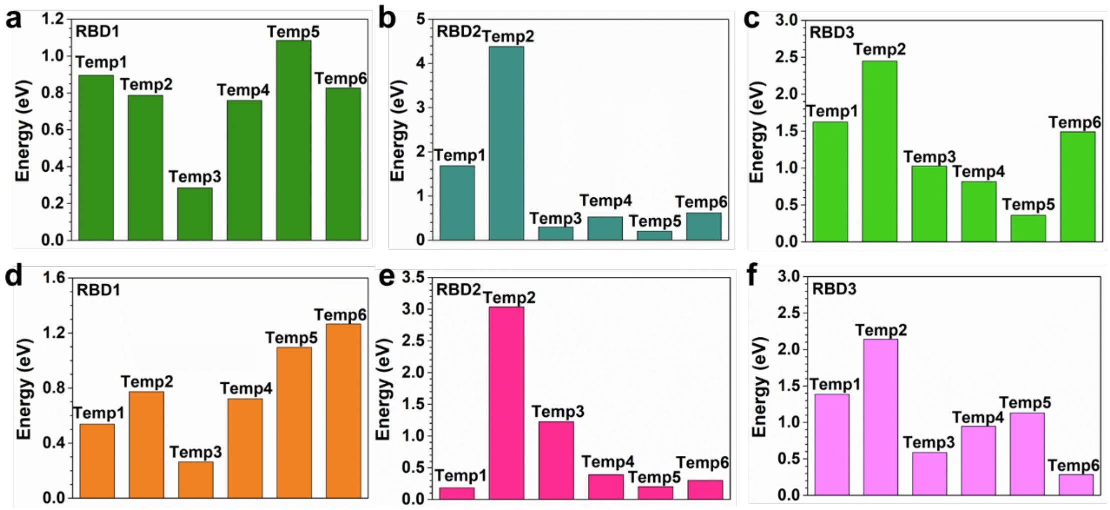

Figure S6. Adsorption energy comparisons. Related to Figure 2.

(a) RBD1, (b) RBD2, and (c) RBD3 segments on Pd (111) surfaces with different binding configurations. Adsorption energy comparison of (d) RBD1, (e) RBD2, and (f) RBD3 segments on Pt (111) surfaces with different binding configurations.

Reference

1. Veeramachaneni, G.K., Thunuguntla, V., Bobbillapati, J., and Bondili, J.S. (2021). Structural and simulation analysis of hotspot residues interactions of SARS-CoV 2 with human ACE2 receptor. J. Biomol. Struct. Dyn. *39*, 4015-4025. 10.1080/07391102.2020.1773318.
2. Huang, Y., Yang, C., Xu, X.F., Xu, W., and Liu, S.W. (2020). Structural and functional properties of SARS-CoV-2 spike protein: potential antivirus drug development for COVID-19. Acta. Pharmacol. Sin. *41*, 1141-1149. 10.1038/s41401-020-0485-4.
